# Oesophageal coins invisible on chest radiography: a case report

**DOI:** 10.1186/s12245-017-0153-8

**Published:** 2017-09-05

**Authors:** Jin Takahashi, Takashi Shiga, Hiraku Funakoshi

**Affiliations:** Department of Emergency and Critical Care Medicine, Tokyo Bay Urayasu Ichikawa Medical Center, 3-4-32, Todaijima, Urayasu, Chiba 279-0001 Japan

**Keywords:** Oesophageal coins, Radiolucent, CT scan

## Abstract

**Background:**

Coins are made of metal, which is generally radiopaque, and so physicians often have the misconception that all coins are detectable by radiography. Here, we report a case of intentionally swallowed coins in the oesophagus of an adult; the coins could not be detected on chest radiography but were detected using computed tomography (CT).

**Case presentation:**

A 46-year-old woman with a history of depression presented to the emergency department after an intentional medication overdose and ingestion of two Japanese 1-yen coins. She complained of persistent retrosternal discomfort. In order to confirm whether the coins were in the oesophagus or trachea, an anteroposterior chest radiograph was obtained; however, no coins were detected. Owing to her persistent symptoms, a chest CT was performed. On the initial CT scan, two 1-yen coins were observed in the oesophagus: one in the middle oesophagus and the other in the lower oesophagus. After the scanning, the patient drank water with permission, but vomited. No coins were found in her vomit, and the symptoms of retrosternal discomfort had completely disappeared. A subsequent CT scan revealed that the two 1-yen coins were in the patient’s stomach.

**Conclusions:**

Japanese 1-yen coins are made of 100% aluminium, which is less radiopaque than the metals that make up coins (nickel, bronze, and lead), and so, they were not visible via chest radiography in our case. Detecting very small or thin radiolucent foreign bodies is not possible using a chest radiograph or contrast oesophagram, but is possible via CT. CT is both increasingly convenient and non-invasive, unlike endoscopy or bronchoscopy, and so, the use of CT scans should be considered in cases of possible radiolucent foreign body ingestion.

## Background

Swallowed foreign bodies can include various objects, with coins being the most common in children. Coins are made of metal, which is generally radiopaque, and so, physicians often have the misconception that all coins are detectable by radiography. Here, we report a case of intentionally swallowed coins in the oesophagus of an adult; the coins could not be detected on chest radiography but were detected using computed tomography (CT).

## Case presentation

A 46-year-old woman with a history of depression presented to the emergency department after an intentional medication overdose and ingestion of two Japanese 1-yen coins. On physical examination, the patient was drowsy owing to an overdose of benzodiazepines and quetiapine; however, her vital signs were within the normal range. There were no abnormal lung sounds and no abdominal tenderness. One hour after her presentation, the patient was alert and oriented, after which she complained of persistent retrosternal discomfort. She did not report dyspnoea or dysphagia.

**Fig. 1:**
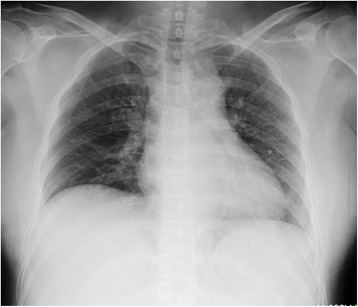
No coins were detectable on the initial anteroposterior chest radiograph

**Fig. 2:**
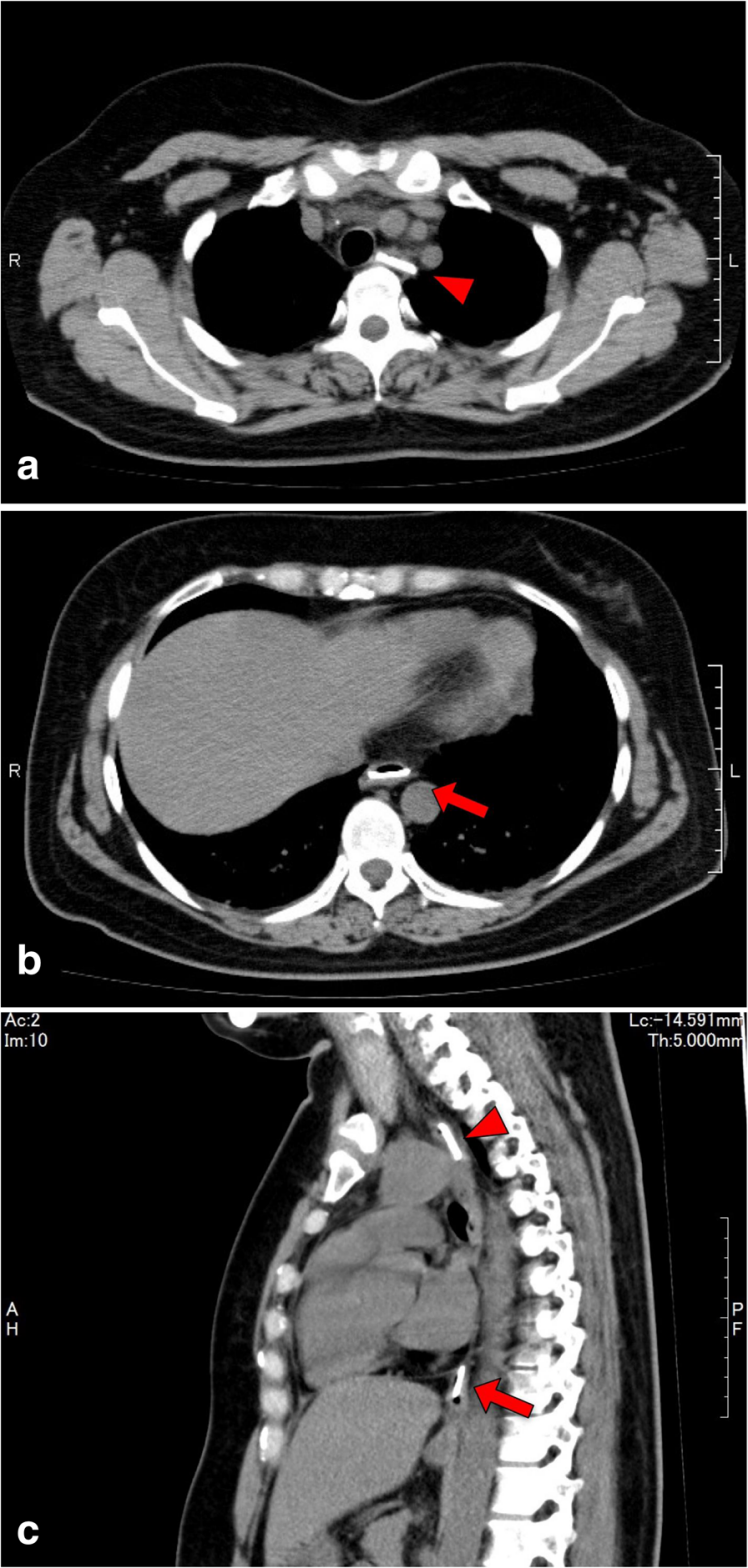
On the initial computed tomography (CT) scan, one coin (*arrowhead*) was found in the middle oesophagus (**a**) and the other (*arrow*) was in the lower oesophagus (**b**). In the sagittal CT multiplanar reconstruction image (**c**), both the coins are observed simultaneously

In order to confirm whether the coins were in the oesophagus or trachea, an anteroposterior chest radiograph was conducted; however, no coins were detected (Fig. 1). The medical team suspected that the coins had already traversed to the stomach or lower gastrointestinal tract, or that she had not swallowed any coins. However, owing to her persistent symptoms, a chest CT was performed. On the initial CT scan, two 1-yen coins were observed in the oesophagus: one in the middle oesophagus and the other in the lower oesophagus (Fig. 2).

After the scan, the patient drank water with permission, but vomited. No coins were found in her vomit, and the symptoms of retrosternal discomfort had completely disappeared. A second CT scan revealed that the two 1-yen coins were in the patient’s stomach. Because there was no need for treatment, the patient was discharged. Two days later, at the follow-up appointment, the patient reported that the coins had been discharged in her faeces.

## Conclusions

Swallowed foreign bodies can consist of various items, including small toys, button batteries, press-through packs, and artificial teeth. Coins are the most common foreign bodies found in children [1]. Coins are made of various metals, such as nickel, bronze, and lead, which are generally radiopaque and can be detected via radiography.

The X-ray absorption of a coin depends on its composition, density, and the atomic number of the metal. Japanese 1-yen coins are made of 100% aluminium, which has an atomic number (*Z*) of 13, compared to nickel and lead, with *Z* values of 28 and 82, respectively. The atomic number for aluminium is between that of the bone (calcium, *Z* = 20) and of the soft tissue (*Z* = 7.5). It is therefore very difficult to distinguish aluminium from other soft tissues [2, 3]. In our case, 1-yen coins could not be detected by chest radiography.

A few studies have reported cases of aluminium-based foreign bodies. Khan reported a case in which a Pakistani 2-rupee coin, also made of aluminium, was faintly visible on a chest radiograph [4]. Kotsenas et al. reported a case in which the aluminium pull tab from a beverage can was aspirated and was not detectable via chest radiography. The pull tab was detected in a chest CT scan 7 months later [2].

Additional reasons for the invisibility of 1-yen coins on the chest radiograph in this particular case include the direction of the X-rays and the location of the coins. In the oesophagus, the coin was located horizontally in relation to the trunk. This horizontal positioning separates the coin from other mediastinal structures or the thoracic spine on a lateral chest radiograph. In addition, the coin could be more easily visualised owing to its thickness in this position.

A contrast oesophagram may also be effective for identifying radiolucent foreign bodies in the oesophagus, while also exposing the patient to less radiation [5]. However, contrast oesophagrams do not possess adequate diagnostic accuracy compared with CT scans, come with a contrast-aspiration risk, and may compromise subsequent endoscopies due to the contrast coating of the foreign body and oesophageal mucosa [6].

It is impossible to detect the presence of a very small or thin radiolucent foreign body on a chest radiograph or contrast oesophagram. For such cases, only a CT scan can detect a radiolucent foreign body in the oesophagus. CT scans are both convenient and non-invasive, unlike endoscopy or bronchoscopy. Therefore, CT scans should be considered in cases of possible radiolucent foreign body ingestion.

## Abbreviations

CT: Computed tomography
Z: Atomic number
